# Supplementary material for: Violence, city size and geographical isolation in African cities
Source: Nat Commun. 2025 Nov 19;16:10049. doi: 10.1038/s41467-025-65728-6 (PMC12630714; doi:10.1038/s41467-025-65728-6)
Supplement: Supplementary file 1 — Supplementary Information [file 41467_2025_65728_MOESM1_ESM.pdf]

Violence, City Size and Geographical Isolation in African Cities

Rafael Prieto-Curiel[1,*] and Ronaldo Menezes[2,3]

[1] *Complexity Science Hub, Metternichgasse 8, 1030 Vienna, Austria*
[2] *BioComplex Laboratory, Computer Science, University of Exeter, EX4 4QJ, Exeter, United Kingdom*
[3] *Computer Science, Federal University of Ceará, 60020-181, Fortaleza, Brazil*

**Corresponding author: r.menezes@exeter.ac.uk*

List of Supplementary Items

Supplementary note 1 - The location of events and the delineation of cities **3**

Supplementary note 2 - Events depending on the distance threshold **6**

Supplementary note 3 - Reported scaling of violence and crime **7**

Supplementary note 4 - Sublinear scaling of violence in African cities **9**

Supplementary note 5 - Are the scaling coefficients the impact of only a few cities? **9**

Supplementary note 6 - Constructing coefficient intervals **12**

Supplementary note 7 - Centrality and isolation **12**

Supplementary note 8 - Income and violence against civilians **13**

Supplementary note 9 - Protests and riots **16**

Supplementary note 1 - The location of events and the delineation of cities

Understanding the dynamics among violent groups is fundamental for assessing their influence on violence and governance. Three primary sources of data could be used to detect the dynamics of urban violence and its links with isolation: the Uppsala Conflict Data Program (UCDP) [1,2], the Global Terrorism Database (GTD) [3] and the Armed Conflict Location and Event Dataset (ACLED) [4]. The datasets are event datasets that are constructed based on media reports, so they have biases and are prone to some errors [5]. First, the UCDP takes media reports but also consults local reports and data from NGOs and international organisations like the United Nations, as well as local events. For example, between 2013 and 2016, approximately 20% of the events in the UCDP were reported from non-media sources, thus improving potential media biases [1]. Secondly, the Global Terrorism Database (GTD) is a database of terrorist incidents since 1970 [3]. GTD data has relevant additional variables to analyse events, such as weapon types, attacks, and targets. Finally, the Armed Conflict Location & Event Data project (ACLED) [4] is the most comprehensive and detailed database of events happening worldwide.

However, there are some differences between the datasets. Most battles in the ACLED dataset are missing events in GTD since they are not considered “terrorism”. Similarly, GTD does not capture protests and riots (also found here to be sublinear in terms of the number of events and casualties). Further, it has been observed that GTD captures roughly half of the terrorist events from ACLED but captures similar patterns and trends [6].

ACLED updates its database frequently, typically with only a few days’ delay between when an event occurs and when it appears in its data. In contrast, UCDP publishes annual reports, resulting in a delay of several months between an event and its inclusion in the dataset. Finally, in terms of volume, ACLED has over 43,000 events in Africa during 2023, whilst UCDP has approximately 4,200 events. Thus, ACLED provides more volume and up-to-date information and it is used in this study.

Detecting which events correspond to an urban agglomeration can be done by computing the intersection between each urban polygon and the coordinates of each event. Data from Africapolis is utilised to ascertain the location and size of over 2,000 cities across the continent, employing a uniform definition of an urban area across all countries [7].

We then consider the delineation of urban agglomerations from Africapolis and, for each city, count the number of reported events and casualties within each polygon [7]. We find that the number of events, casualties, attacks against civilians, and casualties they have created are all sublinear. For events reported since 2015, the number of events has a scaling coefficient $\beta_E \approx \beta_L \approx 2/3$, and similarly for violence against civilians (all coefficients by year are in the SM-A). Thus, looking directly at the events which are recorded within each urban polygon in Africa, we detect a sublinear correlation with city size. People in smaller African cities suffer more violence against civilians than people in larger cities.

The delineation of each city was constructed by looking at a contiguous built-up surface of not more than 200 m of distance. We identify all events that are recorded within its urban delineation and consider the number of events, E_i and the number of casualties

L_i for city i. Then, we consider the expression

$$E_i = \alpha_E P_i^{\beta_E}, \tag{1}$$

where α_E and β_E are two parameters. For each starting year, we compute a Poisson regression to estimate α_E and β_E using [8]. We follow the same strategy for the number of casualties reported within each polygon and obtain α_L and β_L. Results show that β_E and β_L have values far from 1, meaning that the number of events and the number of casualties are both sublinear in Africa, regardless of the starting year considered (Supplementary Table 1).

There are two factors to consider regarding this technique. Firstly, the ACLED data is derived from a wide range of local, national, and international sources in over 75 languages, with efforts to ensure that the most specific possible location and time are recorded. Coordinates are often recorded on strategic locations, natural locations, or neighbourhoods. Importantly, many of the events are coded with geo-referenced coordinates that represent a coarse area rather than a specific location. ACLED includes a variable that refers to the precision at the geographical level. If the source reporting indicates a particular town, and coordinates are available for that town, the highest precision level, 'Geo-precision' code 1, is assigned. When it took place in a small part of a region, the event is coded to a nearby town with 'Geo-precision' code 2. If a larger region is mentioned, often a provincial capital is used with 'Geo-precision' code 3. The geo-precision for protests and riots is very high since more than 95% of events are coded at the most precise level. However, the share is considerably lower for battles, with only around 60% of events recorded with exact location information. Additionally, there are significant geographic differences: in Nigeria, only 55% of violence against civilians events are coded with $geo_precision = 1$, whereas in Zimbabwe, the figure exceeds 95%, reflecting variation in reporting capacity and data quality across contexts. This means that the recorded location of some events might not be precise and is often repeated. In Mogadishu, for example, ACLED has reported more than 1,500 events with more than 4,000 fatalities in the exact same building (27 events in the year 2000 and more than 30 events since the year 2020). The location recorded by ACLED is not a perfect representation of the location where those 1,500 events occurred, but only an approximation that does not work well at the micro level. In Nouakchott, for example, 98.9% of all events registered in the city have been recorded with the exact same location, which corresponds to some building that has no particular relevance in a residential neighbourhood (Supplementary Figure 1).

Secondly, there is also an issue related to the Africapolis urban polygons [7]. The delineation was carried out before 2015, often with aerial pictures that were taken many years before. Thus, the delineation might be considerably outdated, particularly in countries with rapid population growth (such as Somalia, which grew roughly 40% between 2010 and 2020). Urban polygons might be bigger than their Africapolis delineation suggests.

Thus, since the location of events might not be very precise at the local level and the delineation of cities might be outdated, a binary classification of events might be misleading. This might significantly bias the number of events considered for some cities. In Mogadishu, for example, 739 events were reported outside of the city, but within 800 m from the boundary (Supplementary Figure 1 a). This corresponds to more than 8% of the events reported within the limits of Mogadishu. Therefore, if those events were recorded in a slightly different location, or if the city's boundary was constructed based on a more

Supplementary Table 1: Scaling coefficients for the number of events and fatalities considered a different starting year.

	All events		Violence against civilians	
year	β_E	β_F	β_E	β_F
2000	0.6926 ± 0.0011	0.6904 ± 0.0011	0.6422 ± 0.0023	0.6387 ± 0.0023
2001	0.6951 ± 0.0011	0.6878 ± 0.0011	0.641 ± 0.0023	0.6406 ± 0.0023
2002	0.7051 ± 0.0011	0.6904 ± 0.0012	0.6396 ± 0.0024	0.6396 ± 0.0024
2003	0.7047 ± 0.0012	0.7002 ± 0.0012	0.6321 ± 0.0024	0.6372 ± 0.0024
2004	0.7032 ± 0.0012	0.6985 ± 0.0012	0.6279 ± 0.0025	0.6271 ± 0.0025
2005	0.7033 ± 0.0012	0.6973 ± 0.0012	0.6231 ± 0.0026	0.6236 ± 0.0026
2006	0.7016 ± 0.0012	0.6975 ± 0.0012	0.6213 ± 0.0026	0.6197 ± 0.0026
2007	0.6972 ± 0.0012	0.6962 ± 0.0012	0.6152 ± 0.0026	0.6188 ± 0.0026
2008	0.6919 ± 0.0012	0.689 ± 0.0013	0.6113 ± 0.0027	0.6085 ± 0.0027
2009	0.6897 ± 0.0013	0.6837 ± 0.0013	0.6065 ± 0.0027	0.6052 ± 0.0028
2010	0.683 ± 0.0013	0.6826 ± 0.0013	0.5971 ± 0.0028	0.6038 ± 0.0028
2011	0.683 ± 0.0013	0.6736 ± 0.0013	0.6025 ± 0.0029	0.5924 ± 0.0029
2012	0.6796 ± 0.0014	0.672 ± 0.0014	0.5936 ± 0.003	0.5961 ± 0.003
2013	0.672 ± 0.0014	0.6667 ± 0.0014	0.5755 ± 0.0032	0.5858 ± 0.0031
2014	0.6715 ± 0.0015	0.655 ± 0.0015	0.5743 ± 0.0035	0.5659 ± 0.0034
2015	0.6801 ± 0.0017	0.6514 ± 0.0017	0.5993 ± 0.0038	0.5626 ± 0.0037
2016	0.689 ± 0.0018	0.6609 ± 0.0018	0.6047 ± 0.0041	0.5933 ± 0.0041
2017	0.6927 ± 0.0019	0.6711 ± 0.002	0.6039 ± 0.0045	0.5988 ± 0.0044
2018	0.6961 ± 0.0021	0.6683 ± 0.0022	0.5965 ± 0.005	0.5919 ± 0.005
2019	0.696 ± 0.0023	0.6691 ± 0.0024	0.577 ± 0.0057	0.5776 ± 0.0057
2020	0.7018 ± 0.0027	0.6474 ± 0.0029	0.5857 ± 0.0067	0.5417 ± 0.0067
2021	0.732 ± 0.0035	0.6331 ± 0.0039	0.6089 ± 0.009	0.5381 ± 0.0086
2022	0.7138 ± 0.0064	0.3221 ± 0.0103	0.6616 ± 0.0163	0.487 ± 0.0164

recent aerial picture, it is likely that most of those events would be considered part of Mogadishu and suffered by its population.

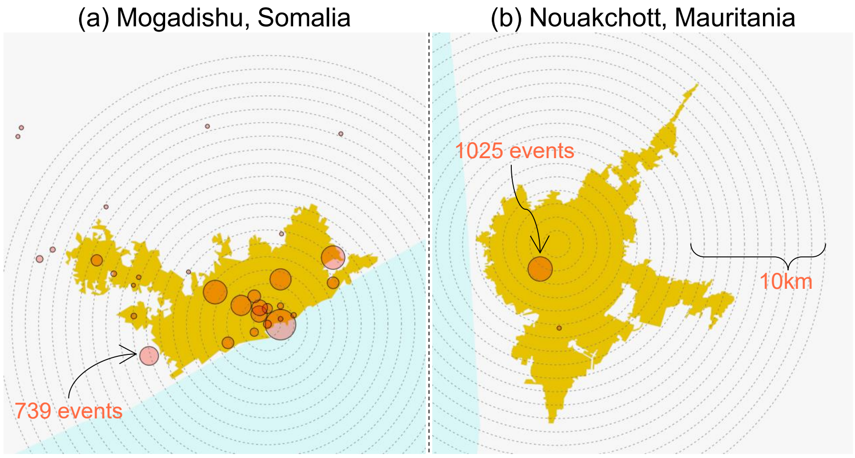

Supplementary Figure 1: Events reported by ACLED between 2000 and 2022 in Mogadishu (a) and Nouakchott (b). The Africapolis urban delineation is the yellow polygon, and the concentric rings are centred in the coordinates of the city centre, and each one has an increment of 1 km radius. The data used in this figure were sourced from Africapolis.org (OECD/SWAC (2024), Africapolis (database), www.africapolis.org, accessed 2024/07/23) and from ACLEDdata.com (ACLED (2025), Acled (database), acleddata.com, accessed 2022/10/25). Of all events reported in Nouakchott, for example, nearly 99% share the same coordinates and roughly 98% have a 'Geo-precision' code 1. Thus, the precise location of those events is not known. Because of the possible misalignment between the spatial polygons and the ACLED events, as in the case of Mogadishu, we constructed a different methodology.

Thus, instead of using only the boundary of each city to classify events as being suffered by its population, we also classify events based on their distance to the city centre.

Supplementary note 2 - Events depending on the distance threshold

An event i is attributed to its nearest city j if the distance to its centre $d_{ij} < \delta$, for a given distance threshold $\delta > 0$. For smaller δ values, fewer events are considered (Supplementary Figure 2). Notably, nearly 2/3 of all events and over half of all casualties occur within 25 km of the cities, despite this area constituting less than 10% of Africa's surface area. Thus, the majority of events transpire either directly within a city or in its proximity [9].

The scaling coefficient for the number of events and number of casualties is sublinear for all values of δ (Supplementary Figure 3 a).

Additionally, data from OpenStreetMap was utilised to digitise the network of all highways across the continent, providing a detailed foundation for analysing the geographic accessibility and connectivity of urban areas [10–12].

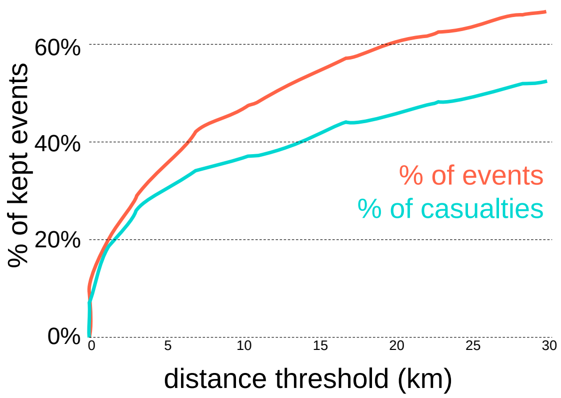

Supplementary Figure 2: Effect of distance on urban rate of events. Percentage of events and fatalities (vertical axis) within the threshold distance δ (horizontal axis) of one of the cities.

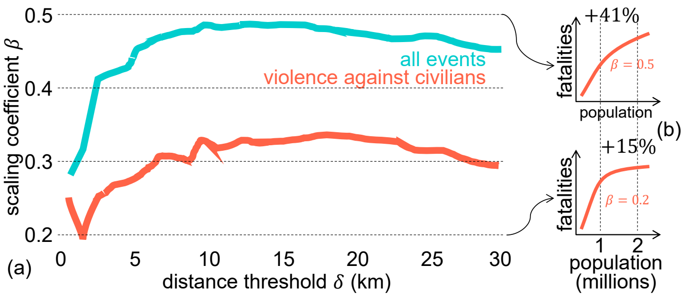

Supplementary Figure 3: Effect of distance on the scaling coefficients. The scaling coefficient β_L represents the number of casualties from all events, and β_V denotes casualties from violence against civilians (vertical axis), varying with the distance threshold δ (horizontal axis) for assigning events to cities. The right side models fatalities based on city size, with two dashed lines indicating casualty numbers for cities of one and two million inhabitants, respectively. Sublinear growth of violence implies that, with $\beta = 0.3$, a city doubling its population sees only a 23% increase in fatalities.

Supplementary note 3 - Reported scaling of violence and crime

Reported scaling for different types of crime (Supplementary Table 2). The list below presents an extensive scaling coefficient between various types of violence and city size, including all known values.

Supplementary Table 2: Observed scaling coefficients for different types of violence in the world. The table is sorted in alphabetical order by the name of the country and divided into two parts, a and b, only for display purposes.

Location	Type of crime	β	Reference
Belgium	Burglary	1.100	13
Belgium	Theft	1.660	13
Brazil	Homicides	1.040	14
Brazil	Homicides	1.150	15
Brazil	Murders	1.150	16
Brazil	Homicides	1.170	17
Brazil	Homicides	1.350	18
Canada	Burglary	0.910	13
Canada	Theft	1.030	13
Colombia	Burglary	0.940	13
Colombia	Homicides	1.060	18
Colombia	Theft	1.260	13
Denmark	Burglary	1.150	13
Denmark	Theft	1.270	13
England and Wales	Violence	1.120	19
England and Wales	Shoplifting	1.260	19
England and Wales	Bike theft	1.273	19
England and Wales	Robbery	1.550	19
France	Theft	1.240	13
France	Burglary	1.290	13
India	Murders and homicides	0.7788	20
India	All crimes	0.8699	20

(a) Part 1

Location	Type of crime	β	Reference
Italy	Burglary	1.090	13
Italy	Theft	1.320	13
Latin America	Homicides	1.100	17
Mexico	Homicides	0.970	17
Mexico	Homicides	1.120	18
Mexico	Theft	1.260	13
South Africa	Burglary	0.910	13
South Africa	Theft	0.970	13
Spain	Theft	1.200	13
United Kingdom	Theft	1.260	13
United Kingdom	Burglary	1.350	13
United States	Sworn police officers	0.820	21
United States	Police budget	0.880	21
United States	Requests for police	0.960	21
United States	Burglary	0.980	13
United States	Burglary	1.010	22
United States	Homicides	1.12	17
United States	Theft	1.130	13
United States	Serious crime	1.160	23
United States	Property crime	1.180	24
United States	All crimes	1.260	21
United States	Robbery	1.350	22
United States	Violent crimes	1.459	24

(b) Part 2

Supplementary note 4 - Sublinear scaling of violence in African cities

To examine the influence of city size on urban lethality, we define the number of casualties in city i as $L_i(\delta)$ and those specifically from violence against civilians as $V_i(\delta)$. We investigate the relationships $L_i \sim P_i^{\beta_L(\delta)}$ and $V_i \sim P_i^{\beta_V(\delta)}$. Formally, this is expressed as:

$$L_i = \alpha_L P_i^{\beta_L}, \tag{2}$$

where α_L and β_L are parameters that vary with the distance threshold δ. Given that event and casualty counts are discrete, a Poisson regression is applied to estimate the values of α_L and β_L.

The number of fatalities per 100,000 inhabitants is proportional to $P_i^{\beta_L - 1}$ once both sides of Equation 2 are divided by city size. Thus, a relevant test is to check whether $\beta_L = 1$. We find that for all values of δ and considering distinct types of events and of starting years, $\hat{\beta_L} < 1$, meaning that small cities have more casualties per 100,000 inhabitants. The same applies to violence against civilians and the obtained values of β_V. The coefficients obtained for different values of δ are in Supplementary Table 3.

Trends changed between 2000 and 2022. The coefficients obtained by considering only events since a specific year are in Supplementary Table 4.

Supplementary note 5 - Are the scaling coefficients the impact of only a few cities?

Some cities with large population sizes (known as dragon-kings), could affect the results when estimating the scaling law parameters [25,26]. This is particularly relevant since the distribution of city sizes is heavy-tailed, meaning there are usually a few cities with millions of people and thousands of smaller areas. As a result, when estimating a scaling coefficient, the values of β may be highly affected by a few large cities. A method devised to detect whether results depend on only a few large cities is through the analysis of repeated sampling [26].

The first strategy is to randomly drop a set of cities from our analysis and re-estimate the coefficients. By systematically removing different subsets of cities, we can assess whether the main conclusions are sensitive to the inclusion of highly violent cities. This method allows us to determine if a few outlier cities primarily drive the findings or if the observed patterns hold more broadly across different urban contexts. Here, we compute the scaling coefficient β, taking all cities at first. Then we randomly drop half of the cities (and their corresponding number of events) and estimate the scaling values of β_s with that sample. This process is then repeated many times (in our case, 1000 times), where we calculate the scaling values of β_s for each iteration. The idea behind this process is that if the scaling coefficient is observed because of a specific large city that causes a considerable bias in the estimation process of β_s, then, that city would be dropped from the estimation process with a 50% chance. Results show that this is not the case (Supplementary Figure 4 - a). The obtained departures from the values of β are always sublinear. The same happens if all events at a distance smaller than $\delta = 20$ km are considered, if only events after 2015, after 2020 or at a distance smaller than $\delta = 10$ km. Additionally, the same sublinear

Supplementary Table 3: Obtained values of α_L, β_L, and of α_V and β_V for some values of δ.

δ	α_L	β_L	α_V	β_V
1	3.5954 ± 0.1486	0.2837 ± 0.0035	2.7564 ± 0.2145	0.2540 ± 0.0064
5	0.5578 ± 0.1153	0.4402 ± 0.0017	1.4398 ± 0.0619	0.2807 ± 0.0035
10	0.3426 ± 0.0405	0.4955 ± 0.0014	0.8308 ± 0.0297	0.3349 ± 0.0029
15	0.3798 ± 0.0062	0.4964 ± 0.0013	0.9488 ± 0.0311	0.3339 ± 0.0026
20	0.46434 ± 0.0072	0.4865 ± 0.0012	0.9584 ± 0.0297	0.3403 ± 0.0025
25	0.5623 ± 0.0085	0.4771 ± 0.0012	1.3283 ± 0.0397	0.3199 ± 0.0024
30	0.7401 ± 0.0108	0.4601 ± 0.0011	1.8392 ± 0.0528	0.2999 ± 0.0024

Supplementary Table 4: Obtained values of α_L, β_L, and of α_V and β_V for some starting year and using $\delta = 10$ km.

Year	α_L	β_L	α_V	β_V
2000	0.3308 ± 0.0059	0.4948 ± 0.0015	0.7843 ± 0.0286	0.3369 ± 0.0029
2001	0.3049 ± 0.0057	0.4944 ± 0.0014	0.7823 ± 0.0288	0.3361 ± 0.0029
2002	0.2418 ± 0.0046	0.5070 ± 0.0015	0.8183 ± 0.0311	0.3281 ± 0.0031
2003	0.2471 ± 0.0048	0.5013 ± 0.0016	0.9604 ± 0.0379	0.3107 ± 0.0032
2004	0.2495 ± 0.0050	0.4971 ± 0.0016	0.9551 ± 0.0390	0.3061 ± 0.0033
2005	0.2414 ± 0.0049	0.4978 ± 0.0016	0.9924 ± 0.0411	0.3010 ± 0.0033
2006	0.2492 ± 0.0052	0.4935 ± 0.0017	1.0129 ± 0.0424	0.2980 ± 0.0034
2007	0.2717 ± 0.0058	0.4839 ± 0.0017	1.0976 ± 0.0469	0.2894 ± 0.0035
2008	0.2877 ± 0.0062	0.4763 ± 0.0017	1.1126 ± 0.0488	0.2850 ± 0.0035
2009	0.2977 ± 0.0066	0.4690 ± 0.0018	1.1616 ± 0.0516	0.2800 ± 0.0036
2010	0.3545 ± 0.0082	0.4508 ± 0.0019	1.3324 ± 0.0607	0.2663 ± 0.0037
2011	0.3682 ± 0.0089	0.4415 ± 0.0020	1.2472 ± 0.0587	0.2667 ± 0.0038
2012	0.3867 ± 0.0097	0.4325 ± 0.0020	1.4692 ± 0.0716	0.2498 ± 0.0039
2013	0.7320 ± 0.0204	0.3685 ± 0.0023	2.4993 ± 0.1351	0.1947 ± 0.0044
2014	0.7651 ± 0.0237	0.3505 ± 0.0025	3.0152 ± 0.1832	0.1648 ± 0.0049
2015	0.6668 ± 0.0234	0.3431 ± 0.0029	2.1797 ± 0.1488	0.1739 ± 0.0055
2016	0.6026 ± 0.0237	0.3349 ± 0.0032	2.2268 ± 0.1665	0.1609 ± 0.0060
2017	0.6141 ± 0.0273	0.3161 ± 0.0036	2.2976 ± 0.1922	0.1438 ± 0.0067
2018	0.6739 ± 0.0344	0.2898 ± 0.0042	2.9320 ± 0.2798	0.1097 ± 0.0077
2019	1.2758 ± 0.0784	0.2158 ± 0.0050	5.1565 ± 0.5751	0.0488 ± 0.0089
2020	1.2931 ± 0.1015	0.1851 ± 0.0064	5.1951 ± 0.7130	0.0308 ± 0.0109
2021	0.9820 ± 0.1211	0.1514 ± 0.0098	2.7164 ± 0.5736	0.0390 ± 0.0161

pattern is observed for the number of fatalities, where the value of β_s is always sublinear for all samples. With the inclusion or exclusion of the dragon king cities in the scaling analysis through sampling, the impact of a few cities on the estimated scaling parameters is detected. If our results are dependent on only a few cities, the scaling coefficients should change rapidly when they are dropped from the analysis.

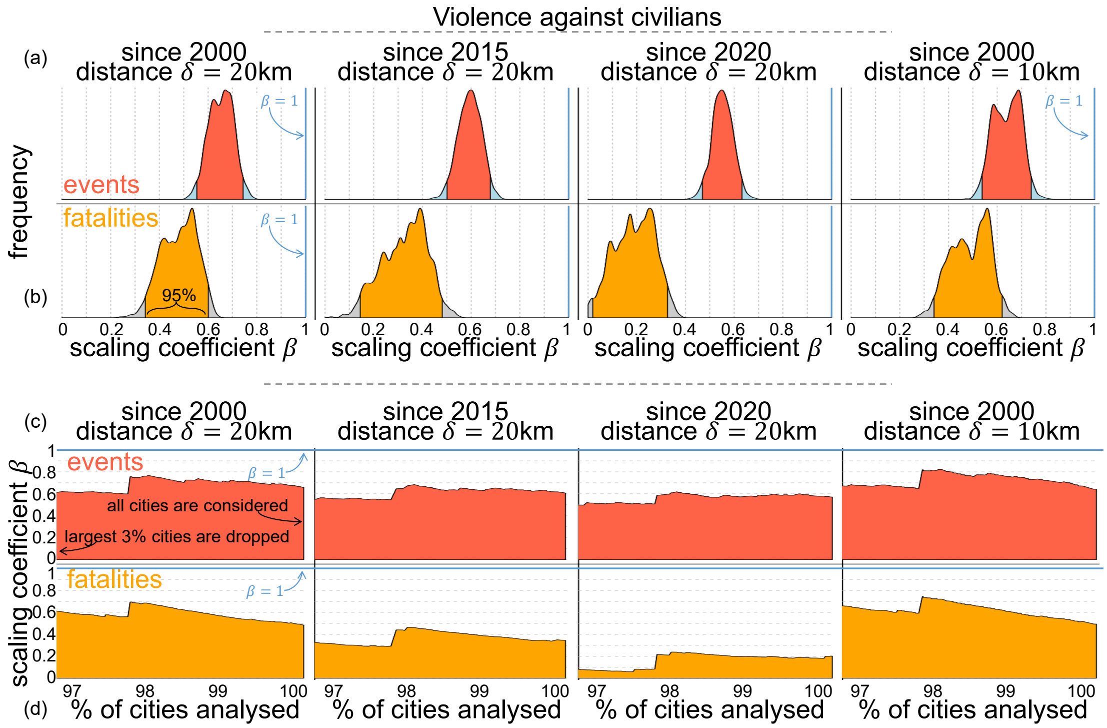

Supplementary Figure 4: Scaling coefficients considering different sets of cities. Scaling coefficient for a resampling process of all cities in Africa when each is kept with a probability of $\pi = 0.5$ and the coefficient β is recomputed (top). Scaling coefficient $\beta(x)$ when the smallest $x\%$ of the cities are considered. When the largest 3% cities are dropped from the analysis, 108 cities are not considered, which is not large in terms of the number of observations, but represent 55% of the urban population considered. Thus, the implication is huge.

Additionally, we test the impact of large cities by considering the observed parameters of the model when only the $x\%$ smallest cities are considered for the analysis. The motivation here is to detect if a specific large city (or set of large cities) is the one causing a deviation from the calculation of the values of β. Formally, cities are sorted according to their size, from the smallest to the largest city on the continent (Cairo, in Egypt), and then consider only the top $x\%$ of the cities from the dataset. Then, for the remaining set of cities, the value of $\beta(x)$ is computed. For $x = 97\%$ we are dropping 108 cities from the analysis, which corresponds to 55% of the urban population on the continent, but with $x = 100\%$ all cities are considered.

When the largest cities are dropped from the analyses, the obtained values of β remain nearly the same (Supplementary Figure 4 - b). The number of events is sublinear for different values of x if all events at a distance smaller than $\delta = 20$ km are considered, if only events after 2015, after 2020 or at a distance smaller than $\delta = 10$ km.

Supplementary note 6 - Constructing coefficient intervals

To investigate whether the observed lethality ratio ϕ_δ results from a limited number of high-casualty events, or "outliers", in isolated cities, we employ a method where, for a given distance threshold δ, we randomly exclude half of the events and calculate the lethality ratio $\phi_\delta^{(1)}$ based on the remaining events. This procedure is replicated 1,000 times, each with a different subset of events, yielding the set $H_\delta = \{\phi_\delta^{(1)}, \phi_\delta^{(2)}, \ldots, \phi_\delta^{(1000)}\}$. We then determine the lower and upper bounds of H_δ. If ϕ_δ were influenced by a few isolated incidents, some iterations of this process would result in $\phi_\delta^{(k)} \approx 1$. Contrary to this hypothesis, our findings do not support such a variation (Supplementary Figure 5).

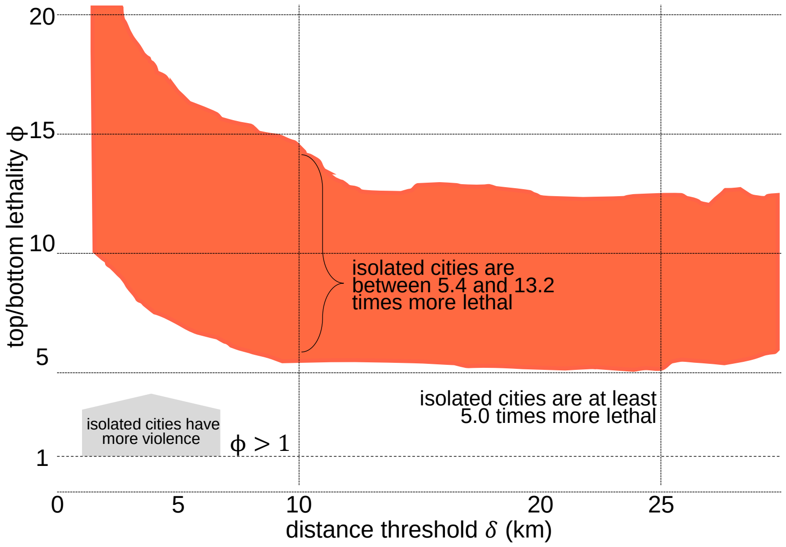

Supplementary Figure 5: Effect of distance on the top-to-bottom lethality. Intervals obtained for the lethality ratio ϕ_δ (vertical axis) depending on the distance threshold δ (horizontal axis).

For all considered values of δ, it is observed that $\phi_\delta^{(k)} > 5$, indicating that isolated cities are at least five times more lethal compared to central cities. Interestingly, the process of randomly eliminating half of the events often results in an increased ratio of top-to-bottom lethality. This pattern suggests that it is the more central cities that occasionally have a few "outliers", while isolated cities exhibit a consistent pattern of violence against their populations, underscoring a systemic issue rather than isolated incidents.

Supplementary note 7 - Centrality and isolation

Primary roads, highways and trunks were obtained from OpenStreetMap [10], downloaded on March 17, 2021, from http://download.geofabrik.de/ in osm.pbf format. We keep motorways, motorway links, primary roads, primary link roads, trunks and trunk links [11,12]. Other road types, including paths and secondary and tertiary roads, are not considered since they are not frequently used for intraurban mobility. The data gives 5.4 million x, y coordinates as a sequence of vertices of different road segments. Curves are defined with a chain of vertices, so straight roads require fewer vertices than winding

roads. We assume that a straight road connects every two consecutive vertices. We keep the physical distance between the two vertices as the length of that segment, using [8,27]. For each road, the sum of all the components gives us the road length. In total, we have 415,231 km of roads on the continent. We construct a simplified road network of the continent by keeping urban agglomerations with more than 100,000 inhabitants, obtained from Africapolis [7], as well as other city attributes, such as its name, country and population in 2015. Each city is then assigned to its closest road coordinates, measured from the centre of the city. For cities with less than 100,000 inhabitants, we measure the distance from the centre to its closest transport node. If a small city is less than 10 km away, we assume that the road passes through that city, so we label the closest transport node as a city node. The set of nodes is composed of 2,162 cities and 5.4 million transport nodes. Edges are added to obtain a connected network. Although the data does not contain those edges, we reason that it is possible to travel (perhaps at a slow speed) between two nearby nodes.

The procedure results in a network with cities, crossings, or terminal vertices as nodes, as well as the travel length and type of road for each edge connecting the nodes. The result is a connected network with 7,361 vertices, where most of them (71%) are road nodes and 9,159 edges. We transform travel lengths into travel times, comparing different types of roads (so that travelling on a highway is faster than travelling through other roads), and because travel time enables us to add an extra cost for crossing an international border. Any edge with its extremes on different countries is a border crossing, so we add 120 minutes for crossing it. We also add extra travel times when crossing any urban agglomeration. We compose it all into some estimated travel time.

The network is used to measure the *degree* of a city and the *centrality*, a weighted node betweenness [28,29]. We use an expression of the gravity model to estimate the number of journeys between each pair of cities, considering city size and the travel time between them. We estimate the number of trips between each pair of cities and then assign that flow through the fastest path in the network. We then add the number of journeys that pass through each node in the network when all pairs of cities are considered. The centrality, C_k, is our estimate of the number of journeys that pass through each city. Small values of C_k suggest that only a few trips pass through that city, and large values indicate that many trips pass through that city, even when they did not start or end there. Thus, a city with large values of C_k has a strategic location in the network that connects distinct clusters or a large city size to compensate.

Supplementary note 8 - Income and violence against civilians

We measure the correlation between the number of events and the number of fatalities in each city and the income per person at the country level. It is not possible to analyse the income at a more refined level since there is limited information regarding the socioeconomic indices of cities. Almost no data at the city level exists beyond some estimates.

At the country level, results show that a country with a higher income per person is correlated with fewer events and a smaller number of fatalities related to violence against civilians (Supplementary Table 5). The coefficients were obtained with a Poisson regression in R [8]. Countries with a lower GDP per person tend to have higher levels of violence,

although this is only a correlation. The causal link between isolation, GDP per person and violence remains unclear. We correlate the number of events and fatalities per city since 2000, using a threshold $\delta = 20$ km.

We test if including the variable *centrality*, quantified as the modelled number of journeys that pass through each city. The variable is our proxy for detecting the levels of isolation of a city. When included in the model, the variable centrality improves the AIC of the models. Results of the model show that by including the variable, a lower Akaike information criterion (AIC) is obtained, meaning that the relative quality of the statistical models is higher, even considering that an extra parameter has to be computed when the variable is included. Thus, isolation at the city level is helpful for correlating the number of events and fatalities at the city level. Additionally, the coefficient of the variable is negative, meaning that there is a correlation between higher centrality (so, lower isolation) and a lower number of events and fatalities.

Supplementary Table 5: Models for the number of events and number of fatalities registered in ACLED at a distance of $\delta = 20$ km from a city centre. We compare two models, one with and one without the variable of centrality. A lower value of the AIC means that by including that variable, the model has a higher relative quality, considering the extra parameter that needs to be calculated.

	events		fatalities	
(Intercept)	4.277^{***}	3.976^{***}	5.522^{***}	5.018^{***}
	(0.062)	(0.064)	(0.044)	(0.045)
log(Pop2015)	0.605^{***}	0.682^{***}	0.483^{***}	0.599^{***}
	(0.003)	(0.004)	(0.002)	(0.003)
log(GDPPP)	-1.178^{***}	-1.094^{***}	-1.033^{***}	-0.902^{***}
	(0.007)	(0.008)	(0.005)	(0.005)
log(centrality)		-0.098^{***}		-0.146^{***}
		(0.003)		(0.002)
AIC	91624.466	90688.481	261932.645	257017.344
Num. obs.	2162	2162	2162	2162

$^{***}p < 0.001$; $^{**}p < 0.01$; $^{*}p < 0.05$

Supplementary note 9 - Protests and riots

The ACLED dataset registered over 100,000 protests and riots between 2000 and 2022 in Africa (Supplementary Table 6).

Supplementary Table 6: Protests and riots in Africa between 2000 and 2022

Type of event	Number of events (2000 - 2022)	Number of casualties (2000 - 2022)
Protests	69,436	4,090
Riots	32,022	18,766
Total	101,458	22,856

The ACLED dataset has registered more than 100,000 protests and riots with nearly 23,000 casualties in Africa between 2000 and 2022.

Nearly 80% of the protests and riots in Africa between January 2000 and October 2022 occurred within 20 km of the centre of a city. Two of three protests and 56% of the riots also happened within 10 km of the centre of a city. Thus, both are typically urban events. Both types usually have a high frequency but tend to have fewer casualties. The average number of fatalities for each event labelled as violence against civilians is 2.6, but for a protest is 0.06, and the average number of fatalities during a riot is 0.59, so they are, in general, less violent types of events.

In terms of the scaling coefficient concerning city size, we find that for protests after January 2010, the number of protests within 20 km of the centre of a city is sublinear with respect to city size (with $\beta = 0.8095 \pm 0.0024$), and it is also sublinear in terms of the casualties related to protests (with $\beta = 0.9638 \pm 0.0099$), although this coefficient is much closer to 1. Regarding the number of riots in Africa after January 2010, there is also a sublinear pattern regarding city size (with $\beta = 0.7366 \pm 0.0039$) and similarly regarding the number of casualties related to riots (with $\beta = 0.9057 \pm 0.0054$).

Supplementary References

1. Sundberg, R. & Melander, E. Introducing theUCDP georeferenced event dataset. *Journal of Peace Research* **50**, 523–532 (2013).

2. Davies, S., Engström, G., Pettersson, T. & Öberg, M. Organized violence 1989–2023, and the prevalence of organized crime groups. *Journal of Peace Research* **61**, 673–693 (2024).

3. Global Terrorism Database 1970 2020, G. National Consortium for the Study of Terrorism and Responses to Terrorism (2022). URL `https://www.start.umd.edu/gtd`.

4. Raleigh, C., Linke, A., Hegre, H. & Karlsen, J. Introducing ACLED: an Armed Conflict Location and Event Dataset: special data feature. *Journal of Peace Research* **47**, 651–660 (2010).

5. Eck, K. In data we trust? a comparison of UCDP GED and ACLED conflict events datasets. *Cooperation and Conflict* **47**, 124–141 (2012).

6. Prieto-Curiel, R., Walther, O. J. & Davies, E. Detecting trends and shocks in terrorist activities. *PloS One* **12**, e0179057 (2023).

7. OECD/SWAC. Africapolis (database). `www.africapolis.org` (2018). Accessed: September 2019.

8. R Core Team. *R: A Language and Environment for Statistical Computing*. R Foundation for Statistical Computing, Vienna, Austria (2024). URL `https://www.R-project.org/`.

9. Radil, S., Walther, O., Dorward, N. & Pflaum, M. Urban-rural geographies of political violence in North and West Africa. *Available at SSRN* (2022).

10. OpenStreetMap contributors. Planet dump retrieved from https://planet.osm.org . `https://www.openstreetmap.org` (2021).

11. Prieto-Curiel, R., Schumann, A., Heo, I. & Heinrigs, P. Detecting cities with high intermediacy in the African urban network. *Computers, Environment and Urban Systems* **98**, 101869 (2022).

12. Prieto-Curiel, R., Heo, I., Schumann, A. & Heinrigs, P. Constructing a simplified interurban road network based on crowdsourced geodata. *MethodsX* **9**, 101845 (2022).

13. Oliveira, M. More crime in cities? on the scaling laws of crime and the inadequacy of per capita rankings—a cross-country study. *Crime Science* **10**, 27 (2021).

14. Ignazzi, C. A. Scaling laws, economic growth, education and crime: evidence from Brazil. *Espace Géographique* **43**, 324–337 (2014).

15. Alves, L. G., Ribeiro, H. V., Lenzi, E. K. & Mendes, R. S. Distance to the scaling law: a useful approach for unveiling relationships between crime and urban metrics. *Plos One* **8**, e69580 (2013).

16. Alves, L. G., Ribeiro, H. V. & Mendes, R. S. Scaling laws in the dynamics of crime growth rate. *Physica A: Statistical Mechanics and its Applications* **392**, 2672–2679 (2013).

17. Bilal, U. *et al.* Scaling of mortality in 742 metropolitan areas of the Americas. *Science Advances* **7**, eabl6325 (2021).

18. Gomez-Lievano, A., Youn, H. & Bettencourt, L. M. The statistics of urban scaling and their connection to Zipf's law. *PloS one* **7**, e40393 (2012).

19. Hanley, Q. S., Lewis, D. & Ribeiro, H. V. Rural to urban population density scaling of crime and property transactions in English and Welsh parliamentary constituencies. *PloS one* **11**, e0149546 (2016).

20. Sahasranaman, A. & Bettencourt, L. M. Urban geography and scaling of contemporary Indian cities. *Journal of the Royal Society Interface* **16**, 20180758 (2019).

21. Banerjee, S., Van Hentenryck, P. & Cebrian, M. Competitive dynamics between criminals and law enforcement explains the super-linear scaling of crime in cities. *Palgrave Communications* **1**, 1–7 (2015).

22. Gomez-Lievano, A., Patterson-Lomba, O. & Hausmann, R. Explaining the prevalence, scaling and variance of urban phenomena. *Nature Human Behaviour* **1**, 0012 (2016).

23. Bettencourt, L. M., Lobo, J., Helbing, D., Kühnert, C. & West, G. B. Growth, innovation, scaling, and the pace of life in cities. *Proceedings of the National Academy of Sciences* **104**, 7301–7306 (2007).

24. Chang, Y. S., Kim, H. E. & Jeon, S. Do larger cities experience lower crime rates? a scaling analysis of 758 cities in the US. *Sustainability* **11**, 3111 (2019).

25. Arcaute, E. *et al.* Constructing cities, deconstructing scaling laws. *Journal of the Royal Society Interface* **12**, 20140745 (2015).

26. Cabrera-Arnau, C. & Bishop, S. R. The effect of dragon-kings on the estimation of scaling law parameters. *Scientific Reports* **10**, 20226 (2020).

27. Hijmans, R. J. *geosphere: Spherical Trigonometry* (2022). URL `https://CRAN.R-project.org/package=geosphere`. R package version 1.5-18.

28. Rodrigue, J.-P. *The geography of transport systems* (Routledge, New York, USA, 2020).

29. Freeman, L. C. Centrality in social networks conceptual clarification. *Social Networks* **1**, 215–239 (1978).
